# Safety and complications of antimicrobial coated compared to conventional intramedullary femoral nails in proximal femoral fractures

**DOI:** 10.1007/s00068-025-02809-7

**Published:** 2025-03-12

**Authors:** Jacob Wiechert, Georg Osterhoff, Christian Kleber, Andreas Höch, Dmitry Notov

**Affiliations:** Klinik und Poliklinik für Orthopädie, Unfallchirurgie und Plastische Chirurgie, Universitätsklinikum Leipzig, Liebigstr. 20, Leipzig, 04103 Germany

**Keywords:** Femur fracture, Intramedullary nail, Coating, Fracture-related infection

## Abstract

**Purpose:**

The aim of this study was to compare one-year mortality and the incidence of complications, particularly fracture-related infections, between patients with proximal femur fractures treated with novel noble metal-coated implants and those treated with uncoated implants, to detect possible effects of the coating on these endpoints.

**Methods:**

*Design*: Retrospective cohort observational study. *Setting*: Level 1 trauma centre. *Patient Selection Criteria*: All patients ≥ 18 years old with proximal femur fracture, who were treated with an intramedullary femur nail between 01.09.2020 and 01.10.2022 were included. The coated group (COATED) included patients who were treated with a coated implant. The control group (CONTROL) was treated with uncoated implants. Pathological fractures were excluded.

**Results:**

188 patients who matched the criteria were included (COATED: 93, CONTROL: 95). There was no significant difference in the one-year mortality or complication rate between the two groups. The fracture-related infection rate did not differ (*p* = 0.31) between both groups. Complications were observed in 59% of all cases and the overall one-year mortality rate was 42%. There was a significant correlation between complication occurrence and hospital stay (*p* < 0.01).

**Conclusion:**

The coated intramedullary nail was similar to the uncoated nail in terms of perioperative complications and 1-year mortality rate. This suggests that the novel coated implant is safe for common clinical use. Further prospective multicentre studies with larger sample sizes are needed to detect a potential impact of coated implants on the incidence of fracture-related infections.

## Introduction

Proximal femur fracture is a common injury with an incidence of 108.7/100,000 in 2019 in Germany [1]. Considering the demographic development in Europe, it is expected that the incidence of proximal femur fractures will rise more than that of other fractures [2]. Only 40 to 60% of the patients reach the mobility they had before the injury [3]. The one-year mortality rate after proximal femur fractures has been reported up to 47% [3]. Early surgery significantly reduces mortality and complication rates [4, 5]. Beside delayed surgery, patient- related risk factors exist, which can increase the mortality rate, e.g. higher age, male gender, lower BMI, or a high Charlson-Comorbidity Index (CCI) [6, 7].

Closed reduction and internal fixation with an intramedullary nail is an accepted treatment for these injuries, offering immediate full weight bearing, lower blood loss and reduced approach morbidity [7]. Fracture-related infections (FRI) belong to the most severe complications. The occurrence of FRI can permanently reduce a patient’s quality of life and increase treatment costs by a factor of up to 6.5 [8]. The documented rate of FRI after femur fractures ranges from 0.8 to 3.2% [9].

Special antibacterial implant coatings have been developed to lower the risk of implant-related infections. Such coatings can consist of antibiotics or ions of noble metals (gold, silver and palladium = Bactiguard (Bactiguard AB, Stockholm, Sweden). This coating with a galvanic effect should impede the attachment of bacteria on surfaces and so prevent biofilm formation [10–12].

The advantages of these coated nails have not been conclusively demonstrated. The aim of this study is to compare the coated implants with uncoated implants in terms of one-year mortality and occurrence of complications, particularly fracture-related infections.

## Methods

In this single-centre retrospective cohort observational study, all patients ≥ 18 years old with a proximal femur fracture, who were treated with an intramedullary femur nail (ZNN CMN (Cephalomedullary Nail) Bactiguard^®^, Zimmer Biomet Holdings Inc., Warsaw, Indiana, USA) and ZNN CMN, Zimmer Biomet (Zimmer Biomet Holdings Inc. Warsaw, Indiana, USA) between 01.09.2020 and 01.10.2022 in a level 1 trauma centre were included. The coated group (COATED) consisted of patients treated with coated implants, while the control group (CONTROL) received uncoated implants. In the first year of the study, all patients received an uncoated implant, in the second year, all patients received a coated implant, there were no other selection criteria. All implants were proximal femur nails. The insertion point was the greater trochanter. The implantation technique did not differ between both types of implants. No open fractures were recorded. Plastic surgery was not needed in any of the cases. Reamer Irrigator Aspirator (RIA) was not used prior to implantation.

Patients with pathological fractures were excluded from the study. Demographics, American Society of Anaesthesiologists score (ASA) and comorbidities for Charlson Comorbidities Index (CCI) were documented [13, 14]. Fractures were classified according to AO Classification 2018 [15].

Complications were divided into implant-related (screw cut-out, loss of reduction, superficial infection, implant-related infection, haematoma, non-union, nail breakage, wound healing difficulties) and general complications (cardiovascular, dermatological, ear-nose-throat infection, gastrointestinal, genitourinary/renal, abnormal lab values (e.g. anaemia), hepatic, musculoskeletal, neurological, psychological, pulmonary). FRI was defined according to the confirmative criteria of Metsemakers et al. (sinus tract, phenotypically same pathogen in at least two deep native samples, positive histology, pus in fracture) [16]. Furthermore, in-house mortality, duration of hospital stay (from admission to discharge) and quality of treatment characteristics (tip apex distance, surgery duration, hospital admission to surgery time, max. fracture gap) were gathered from the digital patient records.

Follow-up was at least 12 months to evaluate one-year mortality. The study was approved by the local ethics committee (049/23 ek).

The data processing and statistical analysis were carried out using IBM SPSS Statistics 29^®^ (IBM Corporation Armonk, NY, USA) and Microsoft Office Excel 2021^®^ (Microsoft Corporation, Redmond, WA, USA). The descriptive statistics were performed by stating the numerical values (n), percentages (%), median (x ~) and interquartile range (IQR) for non-Gaussian distributed data and by means of mean (MW) and standard deviation (±) for Gaussian distributed data. Group comparisons of nominal data were carried out using crosstabs and chi-square tests. The group comparison of Gaussian-distributed data using the t-test and non-Gaussian-distributed data using the Wilcoxon / Mann Whitney U test. The level of statistical significance was defined at a p-value < 0.05. The power and sample size analysis (p value = 0.05, power = 0.8) was performed with the web calculator of the University of Cologne [17].

## Results

188 patients were included in the analysis (COATED: 93, CONTROL: 95). The demographics of both groups are outlined in Table 1 and the results of the endpoint and outcome are displayed in Table 2. Data regarding the one-year survival could be collected from 141 patients (75%).

**Table 1:** Demographics and quality of treatment

	Coated		Control		
	Median	IQR	Median	IQR	p value
Age in years	83	[75.5;90.5]	84	[74;95]	0.86
BMI kg/m^2	24.6	[21.6;27.6]	24.8	[20.9;28.7]	0.4
ASA	3	[2;4]	3	[2;4]	0.33
CCI	5	[4;6]	5	[4;6]	0.75
surgery duration (in min)	78	[33;123]	63	[46;80]	**0.01**
hospital admission to surgery time (in hours)	16	[9;23]	14	[7.5;21.5]	0.27
max. fracture gap (in mm)	2	[0.5;3.5]	2	[0;4]	0.29
Tip apex distance (in mm)					
	female (n)	male (n)	female (n)	male (n)	
Gender	52	41	64	31	0.11

**Fig. 1:**
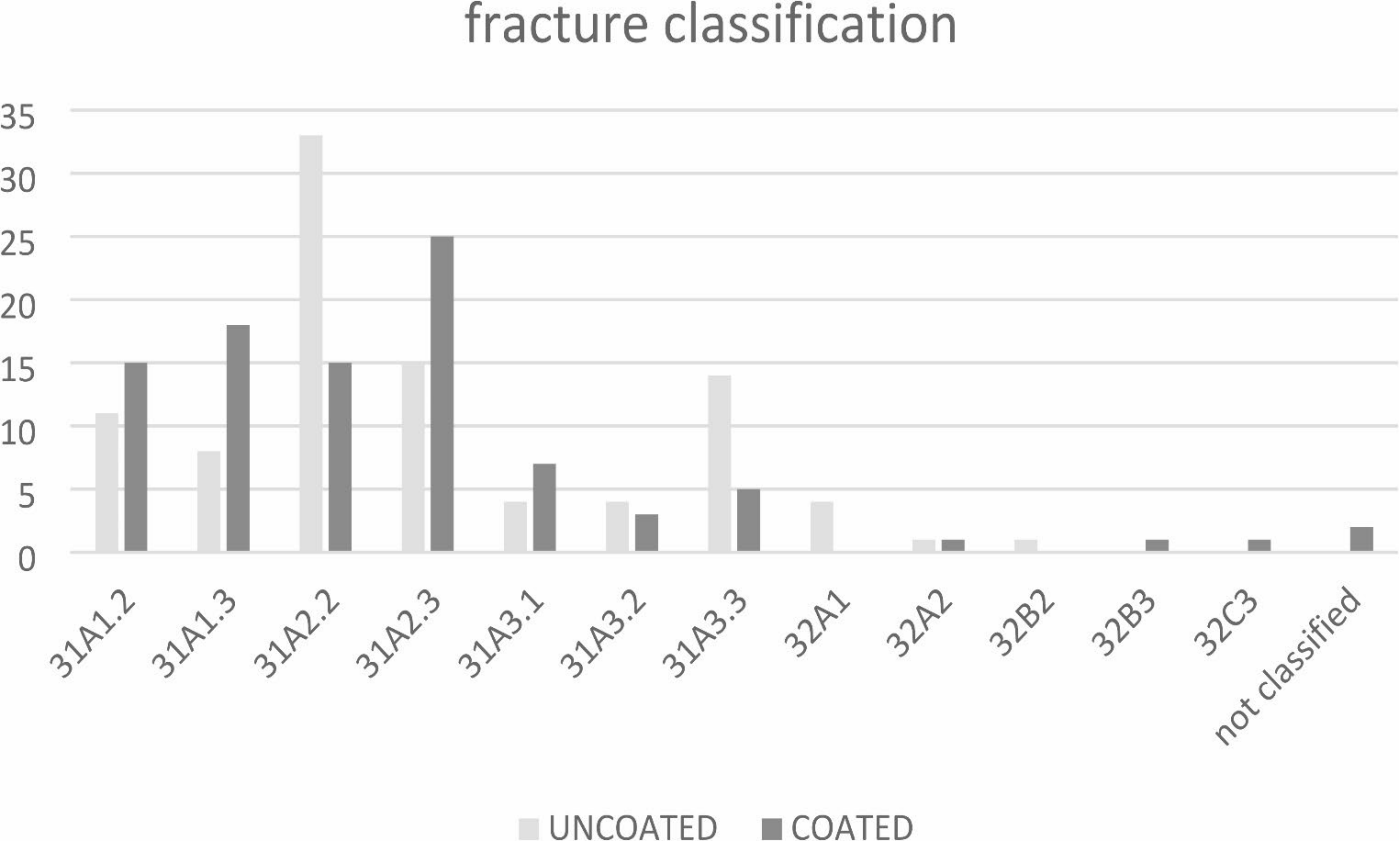
Fracture classification

All included patients had closed fractures. No difference in the distribution of fracture classification (*p* = 0.077) could be observed between both groups. The most common fracture type was 31A2.2 (26%, *n* = 48) followed by 31A2.3 (21%, *n* = 40) as outlined in Fig. 1.

There was no difference in epidemiological and quality of treatment characteristics, besides the surgery duration (*p* < 0.01), which was longer in the COATED group. The tip apex distance was in the median between 10 mm and 20 mm and did not differ between the two groups (*p* = 0,06).

**Table 2:** Endpoints and outcome

	Coated		Control		
	Percentage (number (n))		Percentage (number(n))		p value
Short-term complications					
fracture related infection	3% (*n* = 3)		1% (*n* = 1)		0.31
hospital mortality	12% (*n* = 11)		13% (*n* = 12)		0.87
general complications	56% (*n* = 52)		55% (*n* = 52)		0.87
local complications	11% (*n* = 10)		9% (*n* = 9)		0.77
Long-term complications					
one-year mortality	45% (*n* = 32)		38% (*n* = 26)		0.38
	median	IQR	median	IQR	
hospital stay in days	11	[3.5;18.5]	11	[6;16]	0.43

The rate of implant-related infection did not differ between the two groups (*p* = 0.31): % (*n* = 3) in the coated group and 1% (*n* = 1) in the control group. the overall FRI rate was 2.1%. the power analysis revealed a needed cohort of *n* = 2013, to detect a difference between the two groups with this FRI rate. One-year mortality rate and the incidence of other implant-related complications were also not different between both groups. the demographics of the four cases with FRI are presented in Table 3

**Table 3:** Fracture-related infection cases

Group	Age	gender	Risk factor	Pathogen	Revisions (number)	Implant removed	One-year mortality
Coated	69	female	BMI: 46.2, malignoma, immunosuppression	Enterobacter cloacae, enterococcus faecium, Enterobacter kobei, cutibacterium acnes, bacteroides fragilis	7	yes	Non-survival
Coated	78	male	immunosuppression	staphylococcus aureus	2	no	survival
Coated	84	female	/	staphylococcus epidermidis	2	yes	Non-survival
Control	77	female	Diabetes	escherichia coli	5	yes	survival

**Fig. 2:**
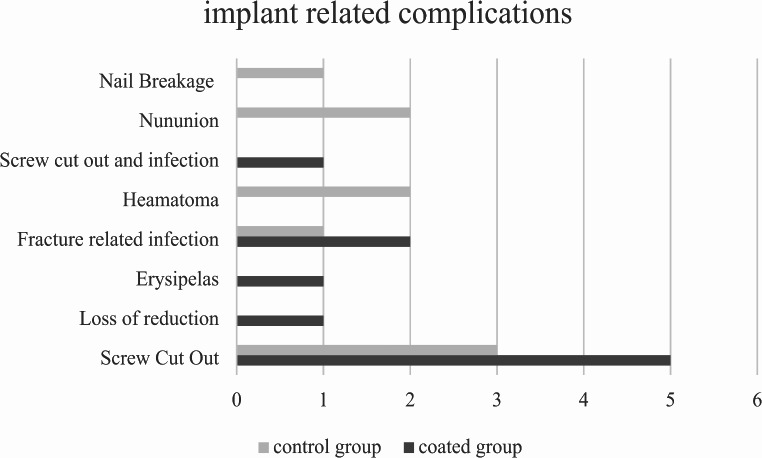
Implant related complications

The most common general complication was anaemia (22%, *n* = 41) followed by urinary tract infection (19%, *n* = 36). Screw cut-out was the most common implant-related complication (4.8%, *n* = 9). The distribution of implant-related complications is outlined in Fig. 2. There was no difference in terms of re-intervention. Revision surgery was required in 16 of the 19 implant-related complications, 8 in the coated nail group and 8 in the uncoated group.

Further analyses showed a significant correlation between complication occurrence and longer hospital stay (*p* < 0.01).

## Discussion

A recognised therapy for patients with a proximal femur fracture is the surgical treatment with intramedullary nailing. The patients with femur fractures are often old [1] and multimorbid [18]. Fracture-related infections are a severe complication with poor prognosis [8]. Bactiguard^®^ technology has proved its efficiency in biofilm and infection reduction in multiple applications. Urinary foley catheters with the Bactiguard^®^ coating could reduce the rate of catheter-associated urinary tract infections by 69%, compared to uncoated catheters [10]. Endotracheal tubes with gold, silver and palladium coating showed less biofilm formation than uncoated endotracheal tubes [11]. The use of noble metal coating on central venous catheters leads to a significantly lower rate of adverse events in comparison to uncoated catheters [12]. Aside from a Malaysian case series, which suggests that intramedullary nails with the Bactiguard^®^ coating can prevent infection and facilitate bone union in patients with open fractures, an uncontrolled case series from Great Britain examined the Bactiguard nail for tibial fractures [19, 20]. No published European data for femur nails with the Bactiguard^®^ coating exists.

The overall FRI rate of 2.1%, which is within the prior published values for proximal femur fractures (0.8 to 3.2%) was observed [9]. In this study, no difference in the FRI rates could be detected between the coated group and the control group. This could be explained by the relatively small population studied (*n* = 188). The power analysis revealed a cohort of *n* = 2013, which would be needed, in order to detect a possible effect of coating on the infection rate. Multicentre studies are necessary to reach cohorts of such size.

A question also arises if coating would be beneficial regarding the infection rates of 2.1% in the studied geriatric population.

It could be rational to examine the possible positive effect of coated implants on FRI rate in patients with a higher risk of infections like open fractures or fixation failure. For example, in patients with nail cut-out, the observed FRI rate was up to 24% [21].

The patients in this study were mainly old with a high number of comorbidities. This is expressed by a median ASA score of 3 and a median CCI of 5. A CCI score of 5 or higher is associated with a one-year mortality rate of 85% [14] and ASA 3 is defined as a patient with severe systemic disease [13]. It was proven in prior studies, that the majority (56.4%) of patients who suffer from a hip fracture are ASA 3 or higher [18]. The results of the current study support these findings.

Dyer et al. reported a one-year mortality rate of proximal femur fractures up to 47% [3]. The mortality rate in this study (42%) is comparable with it. Additionally, the high median age of the patients in this cohort (84 years) is in line with the prior reported findings about patients with proximal femur fracture [8].

Perioperative complications could be observed in 59% of all cases in this study. Mariconda et al. observed an in-hospital general complication rate of 29% [22] and Kenyon-Smith et al. detected a complication rate up to 52% [23]. In comparison to the mentioned studies, the complication rate of this study was in the upper part. ASA and CCI were not assessed in the reference literature, so no comparison was possible. Although the slightly older cohort in this study could have influenced the complication rate.

The implant-related complications were not so common (10% (*n* = 19) with screw cut-out being the most frequent one (4.8%, *n* = 9). Overall, one implant failure (nail breakage) in the control group, was observed. No difference in implant-related complication rates could be observed between both groups (*p* = 0.77) so the coated nail proved to be as stable and safe as the uncoated implant. Complications led to an extension of hospitalisation (*p* < 0.01). This result matches with prior studies [24].

Although the observed complications were mainly not severe (e.g. urinary tract infections) and could be treated without consequential damage in 68%, the pure amount of complication points out, that the treatment of elderly and multimorbid patients with proximal femur fracture needs multidisciplinary cooperation and orthogeriatric comanagement [25].

The strongest limitation of this study is the number of patients included. Because of the low infection rate by closed femur fractures it is difficult to make a statement on this endpoint with only 188 patients. Furthermore, the lack of infection cases hinders the finding of predictive factors for fracture-related infections. Another limitation is that only in 75% of all patients the one-year survival data could be gathered.

This is the first study to examine the orthopaedic implants with noble metal coating with a control group.

Another strength is, that all patients who were treated with these implants in a period of time were included, so that obstacles in routine care for a representative patient collective in Germany could be shown.

## Conclusion

In this study the coated intramedullary nail was similar to the uncoated nail in terms of one-year mortality and appearance of complications, infection rate, and duration of hospital stay. This points out that the coated implant is safe for common clinical use.

Due to the underpowered investigation in terms of the low incidence of FRIs, further studies, with larger populations including patients with a higher risk for FRI (e.g. with open fractures) are needed in order to prove the possible positive effect of noble coating on the infection rate.

## Data Availability

No datasets were generated or analysed during the current study.
